# Coarse-Grained Modeling of Peptide Docking Associated with Large Conformation Transitions of the Binding Protein: Troponin I Fragment–Troponin C System

**DOI:** 10.3390/molecules200610763

**Published:** 2015-06-11

**Authors:** Jacek Wabik, Mateusz Kurcinski, Andrzej Kolinski, Rino Ragno

**Affiliations:** Laboratory of Theory of Biopolymers, Faculty of Chemistry, University of Warsaw, Pasteura 1, 02-093 Warsaw, Poland; E-Mails: jwabik@chem.uw.edu.pl (J.W.); mkurc@chem.uw.edu.pl (M.K.)

**Keywords:** protein docking, flexible docking, coarse-grained modeling, molecular mechanisms

## Abstract

Most of the current docking procedures are focused on fine conformational adjustments of assembled complexes and fail to reproduce large-scale protein motion. In this paper, we test a new modeling approach developed to address this problem. CABS-dock is a versatile and efficient tool for modeling the structure, dynamics and interactions of protein complexes. The docking protocol employs a coarse-grained representation of proteins, a simplified model of interactions and advanced protocols for conformational sampling. CABS-dock is one of the very few tools that allow unrestrained docking with large conformational freedom of the receptor. In an example application we modeled the process of complex assembly between two proteins: Troponin C (TnC) and the N-terminal helix of Troponin I (TnI N-helix), which occurs* in vivo* during muscle contraction. Docking simulations illustrated how the TnC molecule undergoes significant conformational transition on complex formation, a phenomenon that can be modeled only when protein flexibility is properly accounted for. This way our procedure opens up a new possibility for studying mechanisms of protein complex assembly, which may be a supporting tool for rational drug design.

## 1. Introduction

The development of new drugs is one of the most challenging tasks in science today. Combined efforts of the pharmaceutical industry, academic researchers and biotech companies have not only improved the process of drug design, but also contributed to advances in science itself. Computational modeling and simulations have become integral procedures in introducing new drugs to the market and it is safe to assume that the role of theoretical computations in this field will increase in the future [1].

Protein activity in organisms involves interactions with other biomolecules, which makes them perfect targets for rationally designed drugs. After the Human Genome Project [2] was completed, a large number of new drug targets were expected to be found [3]. It soon became evident that a protein’s crystallographic structure alone is, in general, insufficient to understand its function [4]. What in fact is needed, is a complete description of the structure and dynamics of the protein along with its interacting partners. Such a demand from the pharmaceutical industry has recently forced transition in the development of theoretical methods for protein examination: from protein structure prediction to the modeling of whole protein complexes. Moreover, the emphasis in protein modeling is nowadays being put more on obtaining a dynamic picture of a protein system than a simple static structure.

Molecular docking is a technique for predicting the structure of a molecular complex, given the structures of its individual components. There is a great number of software packages available for protein-ligand docking, including DOCK [5], AutoDock [6], HADDOCK (High Ambiguity Driven protein-protein DOCKing) [7], Flex [8], Glide [9], GOLD [10], ZDOCK [11], and others. Docking methods play an important role in drug design, although several important challenges of the docking approach still remain unsolved [12]. Only for a few examples of protein-protein complexes is it possible to obtain high quality models. In most cases docking must be combined with restraints, evolutionary information, or binding site predictions.

It is also problematic to incorporate adequate flexibility to a model of protein docking. In general, when a pair of proteins form a complex, there is a degree of structural rearrangement between unbound and bound conformations. In some cases these changes are marginal and docking can be done using methods based on rigid-body shape complementarity, such as FTDOCK (Fourier Transform Dock) [13] and ZDOCK [11]. These methods exhaustively scan a six-dimensional conformational space of translations and rotations of one molecule* vs.* the other. The rigid-body assumption is naturally a shortcoming for proteins that exhibit large-scale conformational changes on binding. In some cases even small backbone deformations could be essential [14].

The ultimate goal in the development of docking methods is to dock fully flexible molecules without any prior knowledge of the shape and location of the binding site, starting from random conformation of the ligand. This could be achieved in Molecular Dynamics (MD) simulations, but it is highly computationally demanding. MD docking would take an impractical amount of time, even using the most powerful supercomputers. So far, some degree of flexibility only has been handled in a small number of models [15]. For example, ATTRACT employs a coarse-grained model with Normal Mode Analysis [16], whereas the Rosetta’s ‘fold-and-dock’ algorithm uses experimental data: nuclear magnetic resonance shifts and residual dipolar couplings [17].

CABS-dock is a new modeling tool for the efficient modeling of structure and dynamics of protein and peptide complexes. Recently, we demonstrated that the method enables very realistic modeling of fully unrestrained docking of peptide chains with unlimited conformational freedom to a very flexible receptor but restricted to near-native fluctuations [18]. CABS-dock provides a variable, pre-defined level of protein chain flexibility. In the present study, it is used for docking a fully flexible peptide ligand to the receptor with fully flexible linkers between semi-flexible domains. This way large-scale conformational transitions of the receptor can be modeled. Since CABS-dock uses a Monte Carlo dynamics sampling method, this approach can be used not only for predicting native conformations, but also to investigate the mechanism of folding and binding processes. Additionally, we used the CABS-flex [19] tool for investigating near-native fluctuations of a protein complex. It has been shown recently that the results obtained using this tool are consistent with Molecular Dynamics 10 ns simulations [20]. Applications of both modeling tools provide a possibility for the realistic comparison of short-range fluctuations of a receptor molecule alone and a receptor molecule in complex with a peptide.

CABS-dock and CABS-flex are based on the CABS model initially developed for globular protein structure prediction [21]. It has performed exceptionally well in the CASP6 (Critical Assessment of protein Structure Prediction) experiment, where the Kolinski-Bujnicki group was ranked as one of a couple of the best predictors from more than a hundred leading groups [22,23,24,25]. Since then, CABS has been successfully used in a number of other applications, such as protein fragment reconstruction [26], modeling of folding pathways [27,28], protein-peptide docking [29,30,31], modeling of protein flexibility [32], or folding and binding of intrinsically disordered proteins [33]. The main features of the CABS model are briefly described in the Methods Section.

As a proof of concept of fully flexible peptide docking with large conformational changes of the receptor, we study protein-peptide docking in a complex between a fragment of skeletal troponin I and troponin C (pdb code: 1A2X [34]). Troponin is a complex of three proteins: troponin C (TnC), troponin I (TnI) and troponin T (TnT). It plays a crucial role in the regulation of skeletal and cardiac muscle contraction [35]. Troponin binds at regular intervals along the actin polymer on the thin filament. TnT is responsible for binding the whole complex to a tropomyosin molecule. TnC forms the core of the complex: it binds to TnI with variable strength dependent on Ca^2+^ concentration.

The main sites of TnC/TnI interaction are located in the hydrophobic pocket of the N-terminal and C-terminal domains of TnC (TnC N-domain, TnC C-domain). TnC binds two calcium ions with high affinity. Another two calcium ions are bound at a low affinity rate, triggering exposure of the TnC N-domain hydrophobic surface. During the muscle contraction and relaxation cycle Ca^2+^ concentration changes, which in turn triggers conformational transition in the TnC regulatory N-domain. When Ca^2+^ concentration is low, the hydrophobic pocket on the N-domain remains closed, blocking this interaction site for TnI binding. In this conformational state, troponin prevents the approach of myosin heads toward actin [34]. At high Ca^2+^ concentration, the hydrophobic pocket of the N-domain is accessible for TnI binding [36], which may also be co-induced by the cardiac TnI molecule [37].

The second site of interaction between TnC and TnI is located in the hydrophobic pocket of the structural C-domain. The TnI/TnC complex at this end is stable regardless of the calcium ion levels in the sarcoplasm. The interaction of TnC and TnI is important, because it is partly responsible for anchoring troponin to tropomyosin [38]. Mutations that weaken these interactions in cardiac troponin can cause disorders in the cardiac muscle contraction and relaxation cycle [38].

**Figure 1:**
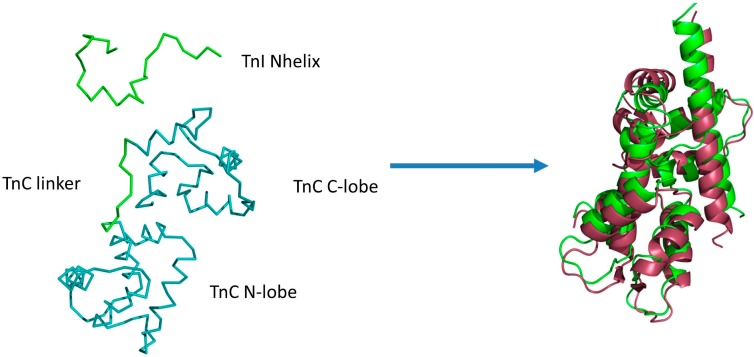
Example initial state of CABS-dock simulations (left side) of the fragment of the skeletal troponin complex in the Ca^2+^-free state of the N-terminal domain of troponin C (TnC N-domain). The N-terminal helix (TnI N-helix) of troponin I and the loop (TnC linker) between TnC domains are treated as fully flexible (green color), allowing unrestricted movement of domains. The remaining part of the structure (two domains in teal color) is fully mobile with domain structures restricted to near-native fluctuations. On the right side the best conformation (RMSD_bound_ = 3.2 Å and RMSD_domains_ = 2.5 Å) from the lowest temperature replica (green color) is shown, aligned with the crystallographic structure (pdb code: 1A2X [34], red color).

In the fragment of the skeletal troponin complex [34], the TnI N-terminal helix interacts not only with the TnC C-domain, but also partly with the TnC N-domain. The TnC domains (Figure 1) are connected by a flexible linker [34,39,40,41,42] and they change their relative positions during complex formation. Movement of such scale cannot be captured by rigid or semi-flexible docking methods.

## 2. Results

**Figure 2:**
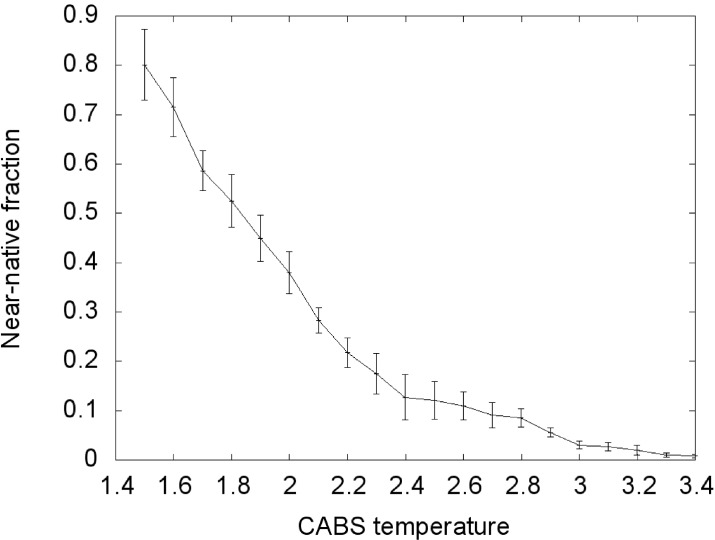
Transition curve of the TnI N-helix (N-terminal helix of troponin I) docking process to skeletal troponin C (pdb code: 1A2X). Conformations for which RMSD (root-mean-square deviation) was below 6 Å were considered near-native. RMSD was calculated on Cα atoms of the troponin C complex with the TnI N-helix* vs.* crystallographic structures.

During the Replica Exchange Monte Carlo (REMC) coarse-grained simulation we obtained a large cluster of near-native conformations in the lowest temperature replica (T = 1.5). From all conformations at this temperature, *ca.* 80% near-native conformations were observed (Figure 2). We assume that the distribution of conformational states in this replica roughly corresponds to distribution at physical room temperature. With increasing temperature the fraction of near-native conformations decreases continuously, reaching about zero with the highest temperature replica. In the T = 2.0 replica, *ca.* 40% near-native conformations were observed, about half of the value seen at the lowest temperature. This is near the transition temperature, where the largest number of transitions between conformational states could be observed.

**Figure 3:**
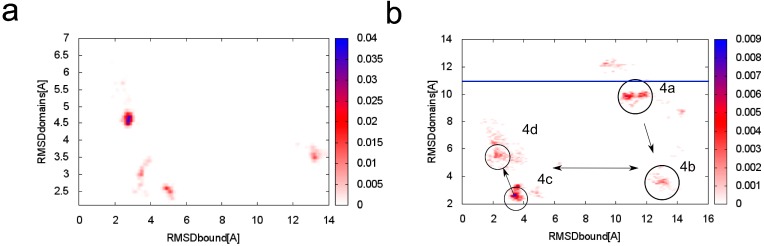
Conformational density map plotted as a function of RMSD_bound_ (root-mean-square deviation) and RMSD_domains_ to the crystallographic structure of skeletal TnC (troponin C) with the N-terminal helix of troponin I (PDB:1A2X [34]) at (**a**) T = 1.5; (**b**) T = 2.0. In the Figure 3b the main clusters are marked with references to Figure 4. The blue horizontal line indicates the RMSD_domains_ value of the crystallographic structure of unbound TnC (PDB: 1TOP [43]).

Because both TnC domains are connected with a flexible linker [34,39,40,41,42], fluctuations of the system are considerable (Figure 3a). In the lowest temperature replica, there are three main clusters of near-native conformations with the main cluster around RMSD_bound_ ~ 3 Å (root-mean-square deviation) and RMSD_domains_ ~ 4.5 Å. 

**Figure 4:**
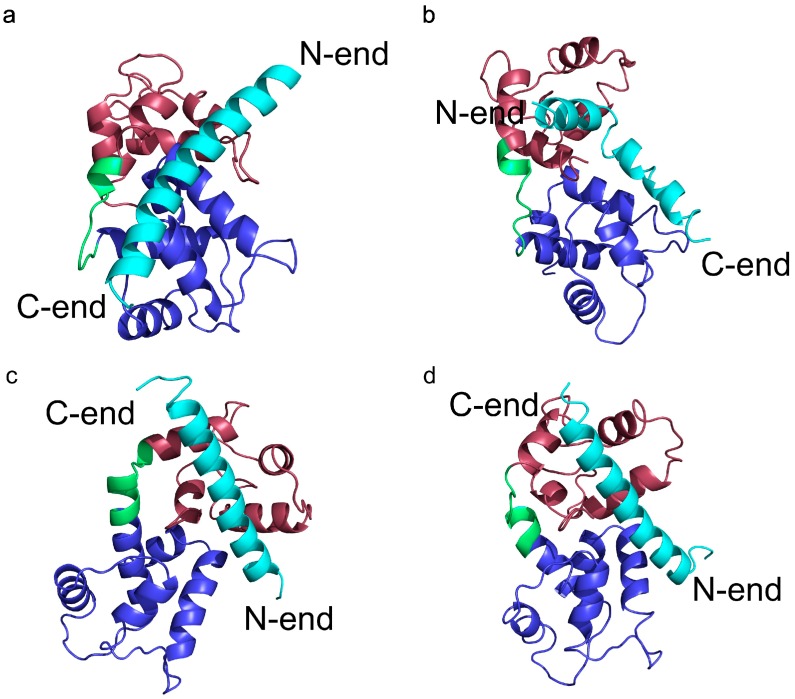
Representative conformations of the four main clusters in the transition temperature replica (T = 2.0) from the CABS-dock simulation of skeletal troponin. It shows transition states in the docking process of the TnI N-helix (N-terminal helix of troponin I) to troponin C. RMSD (root-mean-square deviation) values of the structures are: (**a**) RMSD_bound_ = 11.2 Å and RMSD_domains_ = 9.4 Å; (**b**) RMSD_bound_ = 13.1 Å and RMSD_domains_ = 4.8 Å; (**c**) RMSD_bound_ = 3.6 Å and RMSD_domains_ = 2.6 Å; (**d**) RMSD_bound_ = 3.4 Å and RMSD_domains_ = 4.5 Å.

**Figure 5:**
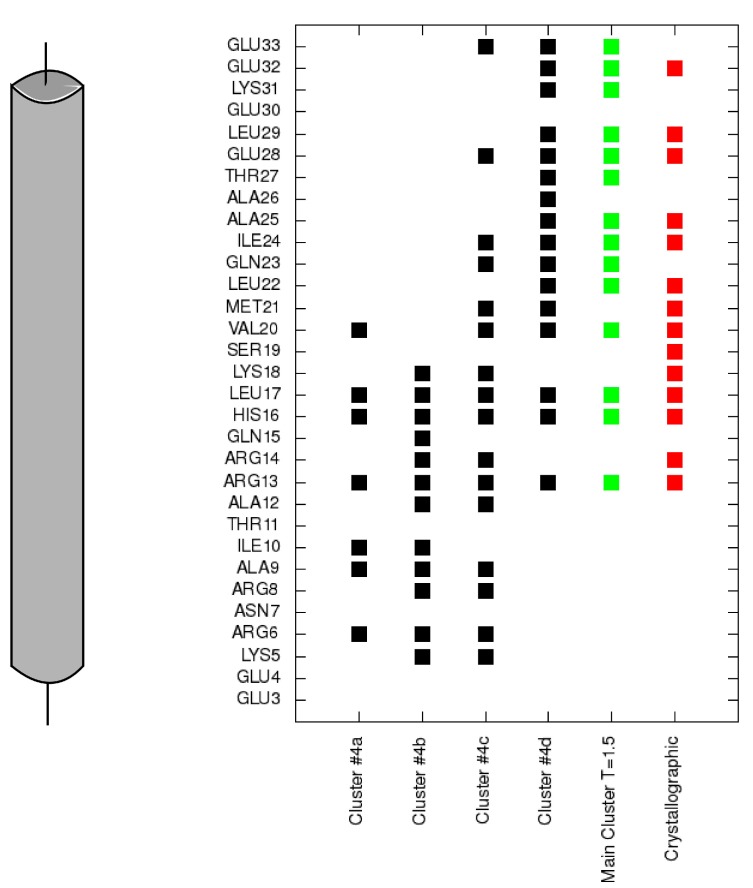
Contacts of each residue of the TnI N-helix (N-terminal helix of troponin I) with the C-terminal domain of troponin C for each cluster representative from Figure 4 (black). Contacts for the largest cluster representative at T = 1.5 are colored green and contacts for the crystallographic structure are colored red.

To analyze transition states observed on protein folding and binding we also isolated clusters of conformations in a 2.0 CABS temperature replica. In Figure 3b we marked four main clusters, which dominate in this replica. In the first cluster representative conformation (Figure 4a) the TnI N-helix is bound to both TnC domains but it is not connected to the hydrophobic pocket of any domain. In Figure 5 it is shown that the TnI N-helix interacts with the amino acids of the TnC C-domain but the TnI N-helix does not have access to the hydrophobic residues of the TnC C-domain. In this unbound state, troponin C is in a collapsed conformation. This transition state of the receptor is not similar to its crystallographic structure in a complex with the TnI N-helix (RMSD_domains_ = 9.4 Å) and it is not present in the lowest temperature replica. In Figure 3b we also notice a bunch of other receptor structures without the ligand inside the hydrophobic pocket. For these conformations RMSD_domains_ is larger than 6 Å. It shows that binding the peptide is essential for stabilizing the receptor conformation. 

In most of the unbound conformations, the peptide has a helical shape. It suggests that the ligand reaches its final structure before binding into the hydrophobic pocket.

In another state (Figure 4b), the TnI N-helix is partially bound to the hydrophobic pocket of the TnC C-domain, but with the helix bound in the opposite direction compared to the native state. In this case, TnI amino acids from the N-terminal part of the helix (residues 8–14) interact with the hydrophobic patch of the TnC C-domain (Figure 5). Such a state is unstable because there are only a few hydrophobic contacts with the TnC C-domain. What is more, the ligand interacts with both TnC domains. We assume that it is one of the alternative states during the folding-binding process. It is noted that interactions between the TnI N-helix with TnC in this conformation also stabilize the arrangement of TnC domains. When the TnI N-helix is inside the hydrophobic pocket of the TnC C-domain, it prevents the TnC domains from approaching each other.

**Figure 6:**
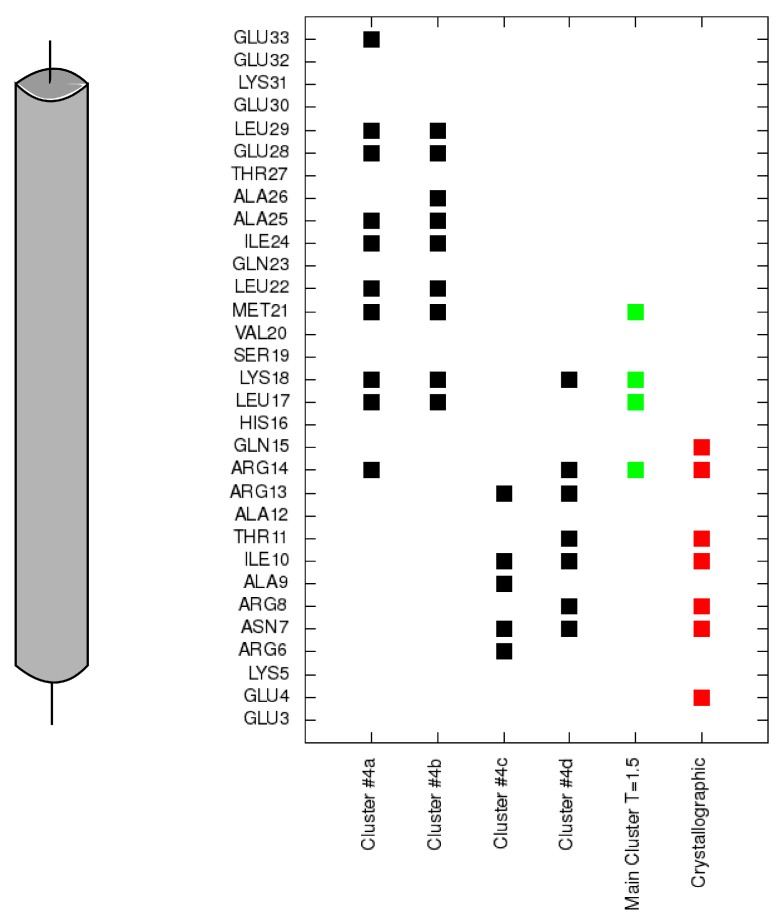
Contacts of each residue of the N-terminal helix of troponin I with the N-terminal domain of troponin C for each cluster representative from Figure 4 (black). Contacts for the largest cluster representative at T = 1.5 are colored green and contacts for the crystallographic structure are colored red.

In conformations 4c and 4d, the TnI N-helix is positioned in the hydrophobic pocket of the TnC C-domain in the proper direction. In both conformations the secondary structure of the helix is stabilized. Nevertheless, in transition state 4c, the TnI N-helix is more shifted in the hydrophobic pocket compared to the crystallographic structure. In structure 4c, the TnI N-helix interacts with the TnC C-domain mainly through TnI residues 12–18. The number of similar conformations with a shifted helix inside the hydrophobic pocket largely decreases in the lowest temperature replica. Structures in the largest cluster in the lowest replica are similar to the structure in Figure 4d. This conformation has the TnI N-helix positioned similarly to the crystallographic structure. The shift of the helix inside the hydrophobic pocket implicates a change in the structure of troponin C and alters non-specific electrostatic interactions (Figure 6) of polar residues (e.g., Arg13, Arg14, Arg8, Asn7, Glu4) of the TnI N-helix with residues of the TnC N-domain. These interactions deviate because of the flexibility of the N-terminal residues of the TnI N-helix and flexibility of the TnC linker. They are not essential to bind the TnI N-helix to TnC but they determine the TnC structure.

**Figure 7:**
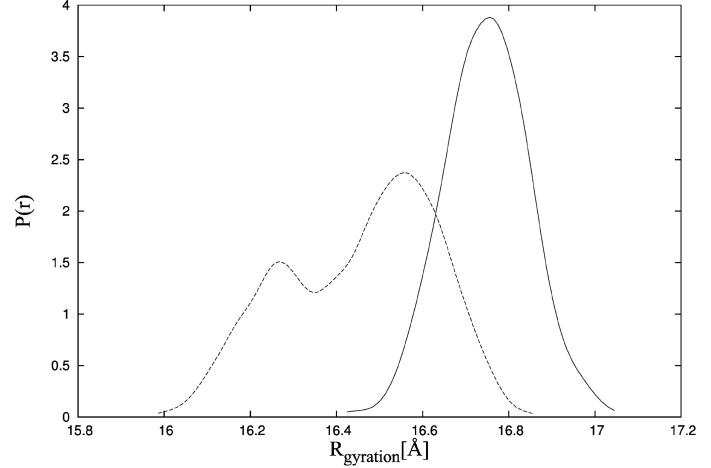
Probability density function of the radius of gyration estimated by the Kernel Density Estimator in the CABS-flex simulations of TnC (troponin C) with the N-terminal helix of troponin I (PDB:1a2x) (solid line) and TnC alone (dotted line).

**Figure 8:**
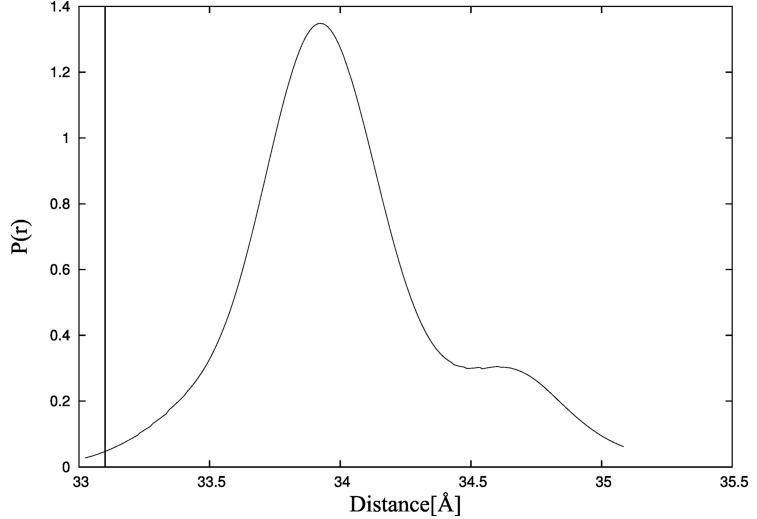
Probability density function of the distance between residues M25-C98 in CABS-flex simulation of troponin C estimated by the Kernel Density Estimator. The vertical line shows the value of the distance measured in fluorescence energy transfer studies [44].

To examine the effect of ligand binding on TnC domain arrangement more precisely, we also use the CABS-flex program. In the first CABS-flex simulation, the starting point is TnC alone in the state from the crystallographic structure of the complex [34]. It is shown that the initial shape of unbound TnC is destabilized (Figure 7, dotted line). The radius of gyration for TnC without the ligand is lower compared to TnC bound to the TnI N-helix (Figure 7, solid line) and fluctuates in the range of 16 Å to 16.8 Å. It is consistent with the experimental data for which the maximum radius of gyration distribution is located near 19 Å [41]. Additionally, the distances between residues M25-C98 for structures obtained during CABS-flex simulation of TnC without the ligand (Figure 8) are consistent with fluorescence energy transfer studies [44].

In the CABS-flex simulation of TnC without the ligand, we do not observe any TnC structures for which the radius of gyration is larger than 17 Å, and it is inconsistent with X-ray solution scattering data [41]. It results from the fact that the CABS-flex program is designed to show local fluctuations only, which occur in the nanosecond time-scale.

## 3. Discussion

The above simulations show that CABS-dock simulation of TnI N-helix flexible docking to TnC converges to the structures, which are near to the crystallographic structure of the complex [34]. The structure with the lowest RMSD (RMSD_bound_ = 3.2 Å and RMSD_domains_ = 2.5 Å) from the lowest temperature replica is shown in Figure 1 and it is aligned with the experimental structure (PDB:1A2X).

The TnC domains in the final complex are close to each other, which enables interactions of the TnI N-helix with both of them. This arrangement is different from the structures of unbound TnC [41,42,43,44].

The crystallographic data of Ca^2+^-saturated troponin C [45] and the crystallographic structure with Ca^2+^ ions bound only to the TnC C-domain [43] indicate that TnC without TnI has extended structure. In this case linker residues between the TnC domains form a helix. Nevertheless, NMR (nuclear magnetic resonance) studies on the TnC structure show that in the Ca^2+^-saturated state TnC forms a whole range of structures [42] and the linker between the domains retains its flexibility. When other kinds of experiments are compared, such as X-ray solution scattering [41] or fluorescence energy transfer [44], it is noted that the dominating state of unbound troponin C is collapsed, with the radius of gyration below 20 Å [41].

**Figure 9:**
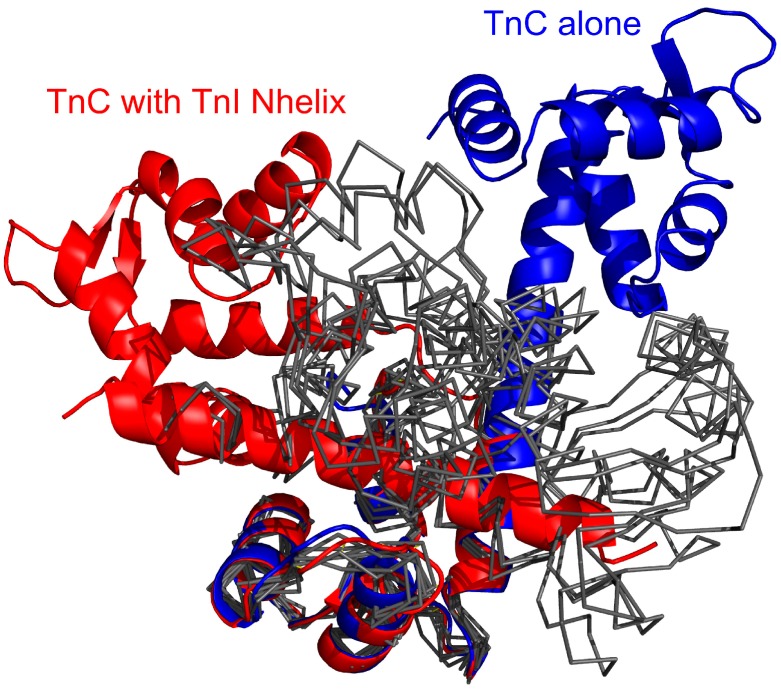
Superposed crystallographic structures of the TnC (troponin C) and troponin I (red color) and unbound TnC [43] (blue) compared with the unbound TnC structures observed in the CABS-dock simulation (black thin lines), where for clarity of conformational changes the TnC C-domain (C-terminal domain of troponin C) only is superposed onto the experimental structures. The unbound structures are cluster representative conformations from one of the temperature replicas (T = 2.0) for which the TnI N-helix (N-terminal helix of troponin I) does not interact with the hydrophobic pocket of the TnC C-domain.

The results of CABS-dock simulations presented in this paper indicate that binding the TnI N-helix is essential to obtain TnC domain arrangement similar to the crystallographic structure of the complex [34]. In Figure 3a it is shown that CABS-dock simulation converges mainly to a cluster of structures with RMSD_domains_ around 4.5 Å. For the structures where the TnI N-helix is not bound to the hydrophobic pocket, TnC has a completely different shape from the crystallographic structure (Figure 3b). In the highest temperature replicas, unbound states dominate. This causes increasing mutual rearrangements of the two receptor domains, but due to the model limitations, the unbound native structure is not clearly represented even at this temperature. Nevertheless, as shown in Figure 9, the structural mobility of the receptor is significant. Experimental structures of unbound skeletal troponin C [42,43,45,46] are also far from the structure of skeletal troponin C with bound TnI [34]. All these findings show that the binding of the TnI N-helix to TnC determines to some extent the arrangement of the TnC domains. This bias to a particular TnC shape is partly caused by non-specific electrostatic interactions of a fragment of the TnI N-helix with the TnC N-domain. The position of the domains is also quite similar to the crystallographic structure when the TnI N-helix is bound in reverse direction in the hydrophobic pocket. Nevertheless, this transition state is not stable because there are not enough hydrophobic contacts to interact with the TnC C-domain and the helical structure of the TnI N-helix is destabilized (Figure 4b and Figure 5).

Finally, it has also been shown that the CABS-dock program is a useful alternative to classical docking approaches, especially for peptide docking to significantly flexible receptors. In this paper we described docking simulations of just one selected system, however many other complexes in which ligand binding causes conformational transitions in receptor structures [47] are already known. Peptide binding to other calmodulin-like proteins is a very important step in calcium signal transduction [48]. Our approach could be used in these similar systems.

## 4. Methods 

### 4.1. CABS Model

The CABS model employs a coarse-grained representation of protein structures and Monte Carlo sampling schemes enabling very fast simulations of the long-time dynamics of protein systems. Its name is an acronym of the first letters of pseudo atoms used to represent a single amino acid, that is: carbon α (CA), carbon β (B) and side chain (S). An additional pseudo atom, defined in the geometrical center of the virtual Cα-Cα bond, is used for defining the main chain hydrogen bonds. CABS uses a simple cubic lattice to store the coordinates of Cα atoms to speed up calculations. The remaining atoms are located off the lattice and “follow” movements of the main chain. The lattice constant is arbitrarily set to 0.61 Å, which translates to a roughly 0.35 Å average error made on casting a protein structure onto the lattice. At these settings there are 800 possible Cα-Cα pseudo bonds, which is a high enough number to prevent any lattice-associated anisotropy.

The knowledge-based force field of the CABS has been derived by statistical analysis of the local regularities observed in protein crystallographic structures, using the Boltzmann inversion. CABS force field consists of five potentials. The first one is the sequence-independent local (close in sequence) terms and biases responsible for stiffening the otherwise very flexible Cα trace. The second potential is sequence-dependent local force derived from the distribution of distances between ith and i + 2nd, i + 3rd and i + 4th Cα atoms. The third one is sequence-dependent pairwise potential for side chain-side chain contact interactions. Attractive part of this potential depends on the mutual orientations of the interacting side-chains and local geometry of the backbone.

Other terms are sequence-independent and mimic excluded volume of the united atoms representing the coarse-grained protein chains. The effects of the solvent and electrostatic interactions are implicitly encoded into the side chain-side chain potential. A detailed description of the force field of the CABS model (also used by CABS-fold [49]) could be found in the literature [21].

Conformational sampling is carried out using Monte Carlo dynamics, controlled by the asymmetric Metropolis scheme. For better sampling coverage of the conformational space, simulations are run parallel in multiple replicas at different temperatures, exchanged between replicas at regular intervals. 

Due to its knowledge-based statistical force field, the temperature of the model is a dimensionless variable proportional to real temperature, although the proportionality parameter needs to be defined at a selected reference state, for instance at a transition observed in the real system in the simulations.

### 4.2. CABS-Dock Procedure

**Figure 10:**
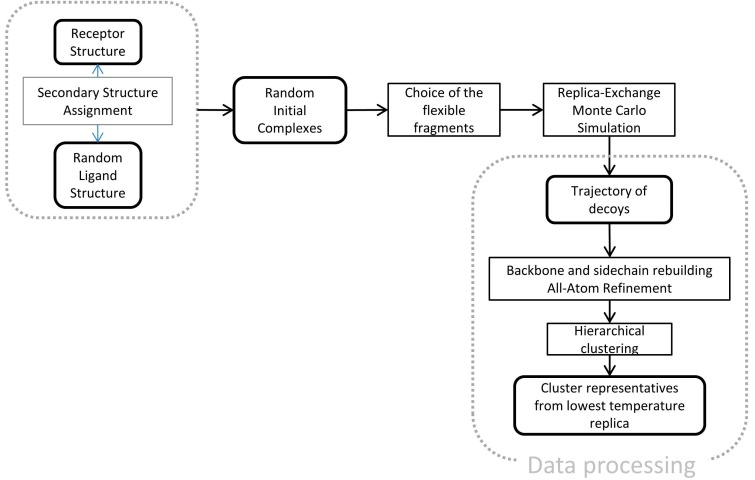
Multiscale docking protocol using the CABS-dock program.

CABS-dock is a multiscale docking protocol built around the CABS model. It consists of the steps showed in Figure 10.

Firstly, the 3D structure of the molecule marked as the receptor (TnC, Figure 1) is projected onto the lattice using least-square fitting. The initial state of the receptor is the same as in the complex with the TnI N-helix (PDB: 1A2X [34]). For the peptide ligand, a TnI N-helix-30 residues (Figure 1) random structure is generated and placed in a random location on the sphere around the geometrical center of the receptor at 20 Å from receptor surface. For each replica, the ligand was positioned at different starting position and conformation.

The next stage is selection of flexible segments present in the docking simulation. CABS-dock allows a variable level of flexibility for different fragments of the receptor. For example, linkers between the domains may be modeled with full flexibility; the active site in a semi-flexible manner (with weak restraints) and the rest of the protein may fluctuate around its native conformation or remain almost completely rigid. Choice of the flexible receptor fragments conditions the results of docking. Larger number of flexible fragments can be put to predict dynamics of the system. In specific cases, it is necessary for successful docking of a peptide to the receptor [18].

In our current work, we applied distance restraints on pairs of atoms within both troponin C domains. It keeps the structural fluctuations of both domains near their individual structures taken from the crystallographic structure of the entire protein (PDB: 1A2X [34]) at the low Ca^2+^ concentration. Structural fluctuations for the lowest temperature replica within both TnC domains are in the range of 1 Å. We did not use any cross-domain distance restraints. TnI (ligand) and the loops between the TnC domains were treated as fully flexible. Such simulation set-ups enabled mutual reorientation of the both TnC domains, indicated by large RMSD (from the crystallographic structure) changes of the whole receptor. 

Replica exchange Monte Carlo simulation is run with 20 replicas in a CABS temperature range of 1.5–3.4. In summary, it gives 2,000,000 MC steps and it took ca. 1.5 day of calculation on the single CPU. Such relatively low computational cost is due to about 4 orders of magnitude faster sampling of protein conformational space by coarse-grained CABS in comparison to all-atom MD simulations.

For further analysis, we take the second half of the CABS trajectory. These models from temperature T = 1.5 and T = 2.0 replicas are clustered using the g_cluster tool from the Gromacs package [50] with the linkage method and 1.5 Å cutoff. Subsequently, the main-chain of each structure is rebuilt and minimized by the MODELLER tool [51].

### 4.3. CABS-Flex Simulations

CABS-flex is a CABS-based protocol applied to observe fluctuations around the near-native state [19]. In this model scaling force field components, distance restraints and time of simulations are adjusted to achieve very good correlation with Molecular Dynamics 10 ns simulations [20]. Here we used it for investigating TnC domain fluctuations. With the aim of performing it, we ran two simulations. In the first one, the starting point was TnC structure complexed with the TnI N-helix (PDB: 1A2X) and the second simulation started from unbound TnC in the same state as in the complex with the TnI N-helix (PDB: 1A2X [34]). For analysis we took the second half of the trajectory.

### 4.4. Data Analysis

Three measures were used to compare the docking results with the crystallographic structure (PDB: 1A2X [34]):

- RMSD_domains_—RMSD calculated on the Cα atoms of both troponin C domains* vs.* crystallographic structure (PDB: 1A2X [34]).
- RMSD_bound_—RMSD calculated on the Cα atoms of the TnC C-terminal domain and the TnI N-terminal helix: residues 3–33.
- RMSD—RMSD calculated on the Cα atoms of TnC and the TnI N-terminal helix: residues 3–33. Conformations for which RMSD was below 6 Å were considered near-native.

To provide receptor-ligand contact analysis, a contact between any two residues was defined as follows: The distance between any two heavy atoms from each residue was smaller than 4.5 Å. In Figure 5 each point shows the contact of a particular TnI residue with any residue from the TnC C-domain. This approach shows which fragment of the TnI N-helix interacts with the TnC C-domain.

In Figure 6 and Figure 7, we estimated the probability density function of radius of gyration and residue-residue distances. Therefore, we used the Kernel Density Estimator. 

The Kernel estimator of an unknown probability density function *f*(*x*) for a given vector of *N* observations *x_i_*, *i*∈[1, *N*] can be defined as follows:

(1) $$\;\;\;{\hat{f}}_{h}\left(x\right)=\frac{1}{Nh}\overset{N}{\underset{i=0}{\sum }}K\left(\frac{\left(x-x_{i}\right)}{h}\right)$$

where *K* is kernel function and *h* is the bandwidth (smoothing parameter). 

In our calculation we used the standard Gaussian function with mean zero and variance 1. For more details see the Bioshell [52] manual.

## 5. Conclusions 

The paper describes the application of the CABS-dock protocol to a difficult problem of protein-peptide complex assembly. Starting from near-native structures of the receptor and completely random structures of the peptide (located at random positions around the receptor structure), we performed REMC simulations of free docking. The docking simulations and the clustering of the manifold of generated conformations led to a well-defined structure of the complex, in good agreement with the available crystallographic structure [34]. Experimental studies also show that the linker between the N-terminal and the C-terminal domain is flexible [34,39,40,41,42]. In accordance with these observations, our simulations revealed significant oscillation of the receptor structure around its native state. It was also shown that the relative position of the TnC domains was dependent on the position of the TnI N-helix.

To investigate the influence of peptide binding on the structure of the whole complex, we also performed CABS-flex simulations, a tool for the computational study of protein domain dynamics. The simulations showed that the binding of the peptide fixed to a large extent the troponin C structure seen in the bound state, while the structures of the receptor without any ligand were closer to the unbound experimental structure of troponin C [41,44] and exhibited higher flexibility.

Finally, let us note that CABS-dock method enables free search (without predefined binding site location) of the peptide docking pathways with properly predefined ranges of the receptor structure fluctuations [18], which gives a possibility of significant domain reorientations for large receptors, as has been shown in this paper. CABS-dock can be particularly useful for studying systems where binding of a peptide induces opening of the binding site of the receptor and therefore is associated with large conformational changes of the receptor [47]. Such free and flexible docking is beyond the ranges of applicability of classical methods.
